# Computer-aided diagnostic accuracy of pulmonary tuberculosis on chest radiography among lower respiratory tract symptoms patients

**DOI:** 10.3389/fpubh.2023.1254658

**Published:** 2023-10-27

**Authors:** Samer Abuzerr, Kate Zinszer

**Affiliations:** ^1^Department of Medical Sciences, University College of Science and Technology, Gaza, Palestine; ^2^School of Public Health, Department of Social and Preventive Medicine, University of Montreal, Montréal, QC, Canada

**Keywords:** computer-aided detection, chest radiography, diagnostic accuracy, GeneXpert, Gaza Strip, pulmonary tuberculosis

## Abstract

Even though the Gaza Strip is a low pulmonary tuberculosis (TB) burden region, it is well-known that TB is primarily a socioeconomic problem associated with overcrowding, poor hygiene, a lack of fresh water, and limited access to healthcare, which is the typical case in the Gaza Strip. Therefore, this study aimed at assessing the accuracy of the automatic software computer-aided detection for tuberculosis (CAD4TB) in diagnosing pulmonary TB on chest radiography and compare the CAD4TB software reading with the results of geneXpert. Using a census sampling method, the study was conducted in radiology departments in the Gaza Strip hospitals between 1 December 2022 and 31 March 2023. A digital X-ray, printer, and online X-ray system backed by CAD4TBv6 software were used to screen patients with lower respiratory tract symptoms. GeneXpert analysis was performed for all patients having a score > 40. A total of 1,237 patients presenting with lower respiratory tract symptoms participated in this current study. Chest X-ray readings showed that 7.8% (*n* = 96) were presumptive for TB. The CAD4TBv6 scores showed that 11.8% (*n* = 146) of recruited patients were presumptive for TB. GeneXpert testing on sputum samples showed that 6.2% (*n* = 77) of those with a score > 40 on CAD4TB were positive for pulmonary TB. Significant differences were found in chest X-ray readings, CAD4TBv6 scores, and GeneXpert results among sociodemographic and health status variables (*P*-value < 0.05). The study showed that the incidence rate of TB in the Gaza Strip is 3.5 per 100,000 population in the Gaza strip. The sensitivity of the CAD4TBv6 score and the symptomatic review for tuberculosis with a threshold score of >40 is 80.2%, and the specificity is 94.0%. The positive Likelihood Ratio is 13.3%, Negative Likelihood Ratio is 0.2 with 7.8% prevalence. Positive Predictive Value is 52.7%, Negative Predictive Value is 98.3%, and accuracy is 92.9%. In a resource-limited country with a high burden of neglected disease, combining chest X-ray readings by CAD4TB and symptomatology is extremely valuable for screening a population at risk. CAD4TB is noticeably more efficient than other methods for TB screening and early diagnosis in people who would otherwise go undetected.

## Introduction

Tuberculosis (TB) stands out as the most widespread illness attributed to a single infectious agent, holding a place among the top 10 leading causes of death worldwide. Although TB can be prevented and treated, it impacts individuals of all age groups. In the year 2019 alone, nearly 10 million people across the globe contracted TB. Among them were 5.6 million males, 3.2 million females, and 1.2 million children (1).

Plain chest radiography remains a crucial tool in identifying early-stage pulmonary tuberculosis (TB) and monitoring the progress of treatments (2). Even when TB patients exhibit no symptoms, chest X-rays (CXRs) exhibit a high degree of sensitivity in detecting abnormalities related to pulmonary TB, particularly when interpreted by proficient radiologists. However, despite this capability, only 7.1 million out of an estimated 10 million TB cases worldwide were actually detected and reported in 2019 (3). Despite a decline in the global incidence rates of TB, these rates still fall short of the targets established by the World Health Organization's (WHO) End TB Strategy (3).

While improvements in digital radiography technology have improved the CXR image quality (4), lack of access to these facilities and skilled radiologists continues to be a problem, especially in underdeveloped regions with a high TB prevalence (5). However, the role of artificial intelligence (AI) in enhancing the accuracy of computer-aided diagnosis for pulmonary tuberculosis (TB) on chest radiography has become increasingly pivotal. AI technologies, such as deep learning algorithms, offer a unique capacity to analyze vast amounts of medical imagery with remarkable precision and speed. In the context of TB diagnosis, AI systems can swiftly and accurately detect subtle abnormalities and patterns on chest X-rays that may elude even skilled human radiologists. By providing reliable and consistent assessments, AI-driven computer-aided diagnosis has the potential to significantly expedite the identification of TB cases, especially in asymptomatic patients, ultimately leading to more timely interventions and improved treatment outcomes. This symbiotic integration of AI with medical diagnostics not only augments the overall diagnostic accuracy but also holds promise for more efficient resource utilization within healthcare systems, thus reinforcing its significance in combating pulmonary tuberculosis on a global scale (6–9). If they perform accurately, these CAD systems may facilitate CXR reading for TB screening and advance the WHO's End TB agenda (3, 10). There are only a few studies in this field, and the majority have methodological flaws, focus on a single CAD program, have scant screening data, or are industry-funded (11, 12).

Furthermore, most studies compared performance against a suboptimal reference standard of a single sputum specimen tested with Xpert MTB/RIF evaluated an online CAD processing system or shared images with the CAD vendors (13, 14). This highlights the need for independent and thorough studies. Offline and multiple AI systems have been the focus of more recent research (15–17), but there are still very few. An international meeting by WHO in 2016 concluded that more data on the effectiveness and application of CAD systems for TB screening were needed (18).

Although the Gaza Strip is a low TB burden region, it is well-known that tuberculosis is primarily a socioeconomic problem associated with overcrowding, poor hygiene, a lack of fresh water, and limited access to health care, which is typical in the Gaza Strip (19, 20). There is a lack of well-organized healthcare infrastructure, which affects the finding and treatment of TB cases, complicated disease control in the Gaza Strip, and a lack of statistics on TB after 2016.

Accordingly, this study aims to evaluate the performance of the automatic software computer-aided detection for tuberculosis (CAD4TBv6) in diagnosing pulmonary TB on chest radiography and compare the CAD4TB software reading with the results of radiologists' reports in the Gaza Strip, Palestine.

## Methods

### Study setting and period

The current study was conducted in radiology departments in the Gaza Strip hospitals between 1 December 2022 and 31 March 2023.

### Study design and study participants

We conducted a cross-sectional study to recruit patients with lower respiratory tract symptoms. Data were collected from respiratory patients referred from the chest department who underwent digital CXRs during the study period using a census sampling method. A digital X-ray, printer, and online X-ray system backed by CAD4TBv6 software were used to screen patients. Patients of both sexes ranging in age from 15 to 80 years were included in this study.

### Sociodemographic and clinical information tool

The acquisition of sociodemographic and clinical information including signs and symptoms of tuberculosis and a history of respiratory diseases was facilitated through the utilization of a meticulously crafted and rigorously validated questionnaire. The questionnaire's design was rooted in a thorough review of pertinent literature and established health assessment frameworks (21–25). This instrument was developed through a systematic process that involved collaboration with domain experts, iterative refinement, and comprehensive pilot testing.

### Assessment of body mass index

Using a measuring rod attached to the balanced beam scale, participants' heights (measured in cm) were recorded to the nearest 0.5 cm while standing barefoot and with their heads up. A common digital weighing scale (SECA, Germany) was used to measure weight (kg). Participants were asked to remove their bulky outerwear before being weighed, and the results were recorded to the nearest 0.1 kg (26).

### CXRs scoring procedures

The obtained CXRs were read within 48 h by a radiologist (certified by the Palestinian board) and classified as normal, probable TB, and non-TB-related. Digital CXRs were scored using CAD4TBv6 (Delft Imaging Systems, Veenendaal, The Netherlands), with scores from 0 to 100 (0 being completely normal and 100 very suggestive of TB) (27).

**Figure 1:**
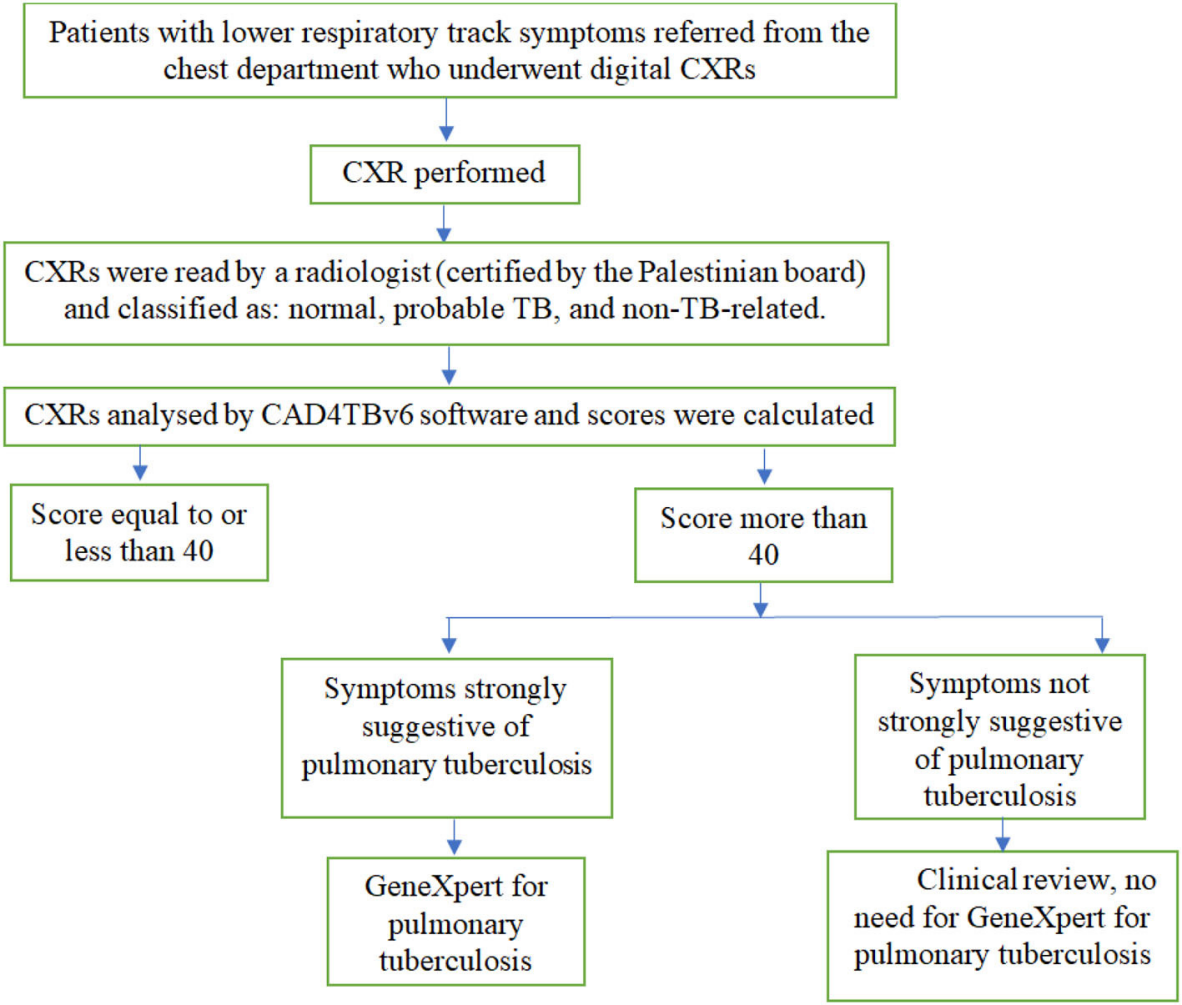
Tuberculosis screening flowchart for patients presenting with lower respiratory tract symptoms.

The analysis was based on identifying aberrant lung field shapes and textures using automatically segmented lung fields. The cutoff point of 40 was chosen (28). Patients who had CXRs images with a score equal to or <40 underwent clinical examination. Whereas, patients who had CXRs with a score higher than 40 were assumed to have tuberculosis. Sputum samples for GeneXpert analysis were obtained only from those with a score > 40 and with symptoms strongly suggestive of pulmonary TB such as hemoptysis, night sweat, weight loss, breathlessness, and fever (Figure 1).

### Statistical analysis

IBM SPSS Statistics for Windows, version 26.0 (IBM Corp., Armonk, NY, USA), was used for statistical analysis. We calculated the frequency and percentage for categorical variables. Continuous variables were analyzed using mean and SD. The chi-square test was used to determine the significant differences between categorical variables. With GeneXpert serving as the reference standard, the following metrics were used for CAD4TB: sensitivity (True Positives x 100/Total Diseased), specificity (True Negatives × 100/Total Non-Diseased), negative predictive value (True Negatives/True Negatives + False Negatives), and positive predictive value (True Positives/True Positives + False Positives).

## Results

### Sociodemographic and health status characteristics, CAD4TBv6 score, and GeneXpert result

A total of 1,237 patients presenting with lower respiratory tract symptoms participated in this current study. More than half of the study participants (56.4%) were males. 45.1% (*n* = 558) of study participants were 41–60 years old, with a mean age ± standard deviation of 47.4 ± 14.2 years. Only 22.1% of study participants (*n* = 273) had a typical BMI of 18.5–24.9, with a mean BMI±std of 30.1 ± 6. 2. 44.9% of the study participants were active tobacco smokers. 75.4% of study participants had a cough of any duration; only 20.0% of them were diabetic patients; 17.5% had hemoptysis; 36.6% had a night sweat; 43.2% experienced weight loss; 79.9% suffered from breathlessness; and 39.7% had a fever. Only 10% of study participants had a past history of TB. Chest X-ray readings showed that 64.8% (*n* = 801) were normal, 27.5% (*n* = 340) were abnormal but not TB and only 7.8% (*n* = 96) were presumptive for TB. There were statistically significant differences in chest X-ray readings between age groups, diabetes status, hemoptysis, night sweats, weight loss, fever, and TB history (*P*-value 0.05).

The CAD4TBv6 scores showed that 11.8% (*n* = 146) of recruited patients were presumptive for TB. Significant differences in CAD4TBv6 scores were discovered across age groups, diabetes status, hemoptysis, night sweats, weight loss, fever, and TB history (*P*-value < 0.05).

GeneXpert testing on sputum samples showed that 6.2% (*n* = 77) of those with a score > 40 on CAD4TB were positive for pulmonary TB. Significant differences were found in GeneXpert results among age groups, hemoptysis, night sweats, weight loss, fever, and history of TB (*P*-value < 0.05).

The study showed that the incidence rate of TB in the Gaza Strip is 3.5 per 100,000 population in the Gaza strip (Table 1).

**Table 1:** Sociodemographic and health status characteristics of patients with presumptive TB by chest x-ray reading, CAD4TB scores, and GeneXpert testing.

	**Chest X-ray reading**					**CAD4TBv6 score**							**GeneXpert testing on sputum samples**			
	**All** ***n*** **(%)**	**Normal** ***n*** **(%)**	**Abnormal, not TB** ***n*** **(%)**	**Suggestive of TB** ***n*** **(%)**	***P*** **-value** ^*^	**All** ***n*** **(%)**	≤ **20** ***n*** **(%)**	**21–40** ***n*** **(%)**	**41–60** ***n*** **(%)**	**61–80** ***n*** **(%)**	>**81** ***n*** **(%)**	***P*** **-value** ^*^	**All** ***n*** **(%)**	**Positive**	**Negative**	***P*** **-value** ^*^
**Gender**																
Male	698 (56.4)	446 (57.1)	190 (55.9)	62 (64.6)	0.244	698 (56.4)	429 (55.3)	180 (57.1)	20 (55.6)	12 (54.5)	57 (54.5)	0.555	62 (64.6)	48 (62.3)	14 (73.7)	0.164
Female	539 (43.6)	335 (42.9)	150 (44.1)	34 (35.4)		539 (43.6)	347 (44.7)	135 (42.9)	16 (44.4)	10 (45.5)	31 (35.2)		34 (35.4)	29 (37.7)	5 (26.3)	
**Age (mean** ±**std** = **47.4** ±**14.2)**																
<25	83 (6.7)	65 (8.1)	18 (5.3)	0 (0.0)	0.001	83 (6.7)	64 (8.2)	18 (5.7)	1 (2.8)	0.0 (0.0)	0.0 (0.0)	0.001	0 (0.0)	0 (0.0)	0 (0.0)	0.001
25–40	330 (26.7)	236 (29.5)	78 (22.9)	16 (16.7)		330 (26.7)	225 (29.0)	78 (24.8)	6 (16.7)	5 (22.7)	16 (18.2)		16 (16.7)	13 (16.9)	3 (15.8)	
41–60	558 (45.1)	355 (44.3)	139 (40.9)	64 (66.7)		558 (45.1)	345 (44.5)	126 (40.0)	16 (44.4)	14 (63.6)	57 (64.8)		64 (66.7)	52 (67.5)	12 (63.2)	
>60	266 (21.5)	145 (18.1)	105 (30.9)	16 (16.7)		266 (21.5)	142 (18.3)	93 (29.5)	13 (36.1)	3 (13.6)	15 (17.0)		16 (16.7)	12 (15.6)	4 (21.1)	
**BMI (mean** ±**std** = **30.1** ±**6.2)**																
<18.5	7 (0.6)	4 (0.5)	3 (0.9)	0 (0.0)	0.949	7 (0.6)	4 (0.5)	3 (0.1)	0.0 (0.0)	0.0 (0.0)	0.0 (0.0)	0.999	0 (0.0)	0 (0.0)	0 (0.0)	0.481
18.5–24.9	273 (22.1)	177 (22.1)	71 (20.9)	25 (26.0)		273 (22.1)	173 (22.3)	65 (20.6)	7 (19.4)	5 (22.7)	23 (26.1)		25 (26.0)	22 (28.6)	3 (15.8)	
25–29.9	383 (31.1)	249 (31.1)	103 (30.3)	31 (32.3)		383 (31.0)	243 (31.3)	94 (29.8)	12 (33.3)	7 (31.8)	27 (30.7)		31 (32.3)	23 (29.9)	8 (42.1)	
30–34.9	317 (25.6)	203 (35.3)	90 (26.5)	24 (25.0)		317 (25.6)	197 (25.4)	82 (26.0)	9 (25.0)	7 (31.8)	22 (25.0)		24 (25.0)	21 (27.3)	3 (15.8)	
35–40	165 (13.3)	106 (13.2)	47 (13.8)	12 (12.5)		165 (13.3)	99 (12.8)	47 (14.9)	5 (13.9)	2 (9.1)	12 (13.6)		12 (12.5)	7 (9.1)	5 (26.3)	
>40	92 (7.4)	62 (7.7)	26 (7.6)	4 (4.2)		92 (7.4)	60 (7.7)	24 (7.6)	3 (8.3)	1 (4.5)	4 (4.5)		4 (4.2)	4 (5.2)	0 (0.0)	
**Active tobacco smoker**																
Yes	556 (44.9)	346 (43.2)	160 (47.1)	50 (52.1)	0.167	556 (44.9)	334 (43.0)	149 (47.3)	16 (44.4)	12 (54.5)	45 (51.1)	0.402	50 (52.1)	37 (48.1)	13 (68.4)	0.096
No	681 (55.1)	455 (56.8)	180 (52.9)	46 (47.9)		681 (55.1)	442 (57.0)	166 (52.7)	20 (55.6)	10 (45.5)	43 (48.9)		46 (47.9)	40 (51.9)	6 (31.6)	
**Cough of any duration**																
Yes	933 (75.4)	594 (74.2)	260 (76.5)	79 (82.3)	0.189	933 (75.4)	575 (74.1)	244 (77.5)	24 (66.7)	19 (86.4)	71 (80.7)	0.226	79 (82.3)	62 (80.5)	17 (89.5)	0.191
No	304 (24.6)	207 (25.8)	80 (23.5)	17 (17.7)		304 (24.6)	201 (25.9)	71 (22.5)	12 (33.3)	3 (13.6)	17 (19.3)		17 (17.7)	15 (19.5)	2 (10.5)	
**Diabetes**																
Yes	248 (20.0)	133 (16.6)	92 (27.1)	23 (24.0)	0.001	248 (20.0)	131 (16.9)	81 (25.7)	12 (33.3)	5 (22.7)	19 (21.6)	0.004	23 (24.0)	16 (20.8)	7 (36.8)	0.179
No	989 (80.0)	668 (83.4)	248 (72.9)	73 (76.0)		989 (80.0)	645 (83.1)	234 (74.3)	24 (66.7)	17 (77.3)	69 (78.4)		73 (76.0)	61 (79.2)	12 (63.2)	
**Hemoptysis**																
Yes	216 (17.5)	2 (0.2)	119 (35.0)	95 (99.0)	0.001	216 (17.5)	2 (0.3)	101 (32.1)	13 (36.1)	13 (59.1)	87 (98.9)	0.001	95 (99.0)	76 (98.7)	19 (100.0)	0.001
No	1,021 (82.5)	799 (99.8)	221 (65.0)	1 (1.0)		1,021 (82.5)	774 (99.7)	214 (67.9)	23 (63.9)	9 (40.9)	1 (1.1)		1 (0.1)	1 (1.3)	0 (0.0)	
**Night sweat**																
Yes	453 (36.6)	175 (21.8)	185 (54.1)	95 (99.0)	0.001	453 (36.6)	169 (21.8)	163 (51.7)	18 (50.0)	16 (72.7)	87 (98.9)	0.001	95 (99.0)	76 (98.7)	19 (100.0)	0.001
No	784 (63.4)	626 (78.2)	157 (45.9)	1 (1.0)		784 (63.4)	607 (78.2)	152 (48.3)	18 (50.0)	6 (27.3)	1 (1.1)		1 (0.1)	1 (1.3)	0 (0.0)	
**Weight loss**																
Yes	535 (43.2)	240 (30.0)	200 (58.8)	95 (99.0)	0.001	535 (43.2)	233 (30.0)	176 (55.9)	23 (63.9)	16 (72.7)	87 (98.9)	0.001	95 (99.0)	76 (98.7)	19 (100.0)	0.001
No	702 (56.8)	561 (70.0)	140 (41.2)	1 (1.0)		702 (56.8)	543 (70.0)	139 (44.1)	13 (36.1)	6 (27.3)	1 (1.1)		1 (0.1)	1 (1.3)	0 (0.0)	
**Breathlessness**																
Yes	988 (79.9)	625 (87.0)	280 (82.4)	83 (86.5)	0.061	988 (79.9)	604 (77.8)	263 (83.5)	27 (75.0)	19 (86.4)	75 (85.2)	0.123	83 (86.5)	66 (85.7)	17 (89.5)	0.230
No	249 (20.1)	176 (22.9)	60 (17.6)	13 (13.5)		249 (20.1)	172 (22.2)	52 (16.5)	9 (25.0)	3 (13.6)	13 (14.8)		13 (13.5)	11 (14.3)	2 (10.5)	
**Fever**																
Yes	491 (39.7)	202 (25.2)	193 (56.8)	96 (1000)	0.001	491 (39.7)	193 (24.9)	176 (55.9)	18 (50.0)	16 (72.7)	88 (100.0)	0.001	96 (100.0)	77 (100.0)	19 (100.0)	0.001
No	746 (60.3)	599 (74.8)	147 (43.2)	0 (0.0)		746 (60.3)	583 (75.1)	139 (44.1)	18 (50.0)	6 (27.3)	0 (0.0)		0 (0.0)	0 (0.0)	0 (0.0)	
**Past history of TB**																
Yes	124 (10.0)	0 (0.0)	74 (21.8)	50 (52.1)	0.001	124 (10.0)	0 (0.0)	62 (19.7)	8 (22.2)	5 (22.7)	49 (62.8)	0.001	50 (52.1)	41 (53.2)	9 (47.4)	0.001
No	1,113 (90.0)	801 (100.0)	266 (78.2)	46 (47.9)		1,113 (90.0)	776 (100.0)	253 (80.3)	28 (77.8)	17 (77.3)	29 (37.2)		46 (47.9)	36 (46.8)	10 (52.6)	

^*^P-value was calculated using the chi-squared test.

The sensitivity of the CAD4TBv6 score and the symptomatic review for tuberculosis with a threshold score of >40 is 80.2%, and the specificity is 94.0%. The positive likelihood ratio is 13.3%, negative likelihood ratio is 0.2, with a 7.8% prevalence. The positive predictive value is 52.7%, the negative predictive value is 98.3%, and the accuracy is 92.9% (Table 2).

**Table 2:** Estimated diagnostic accuracy of CAD4TBv6 software in diagnosing pulmonary TB on chest radiography with a threshold score of >40.

**Test**	**Pulmonary TB**				
	**Present**	***n***	**Absent**	***n***	**Total**
Positive	True positive	77	False positive	69	146
Negative	True negative	19	True negative	1,072	1,091
**Total**		96		1,141	
**Statistics**	**Value**	**95% CI**			
		**Upper**		**Lower**	
Sensitivity	80.2%	70.8%		89.6%	
Specificity	94.0%	92.4%		95.3%	
Positive likelihood ratio	13.3	10.3%		17.0%	
Negative likelihood ratio	0.2	0.1%		0.3%	
TB prevalence^*^	7.8%	6.3%		9.4%	
Positive predictive value^*^	52.7%	46.5%		58.9%	
Negative predictive value^*^	98.3%	97.4%		98.8%	
Accuracy^*^	92.9%	91.3		94.3%	

^*^These values are dependent on disease prevalence. CI, confidence interval.

### CAD4TB analysis

**Figure 2:**
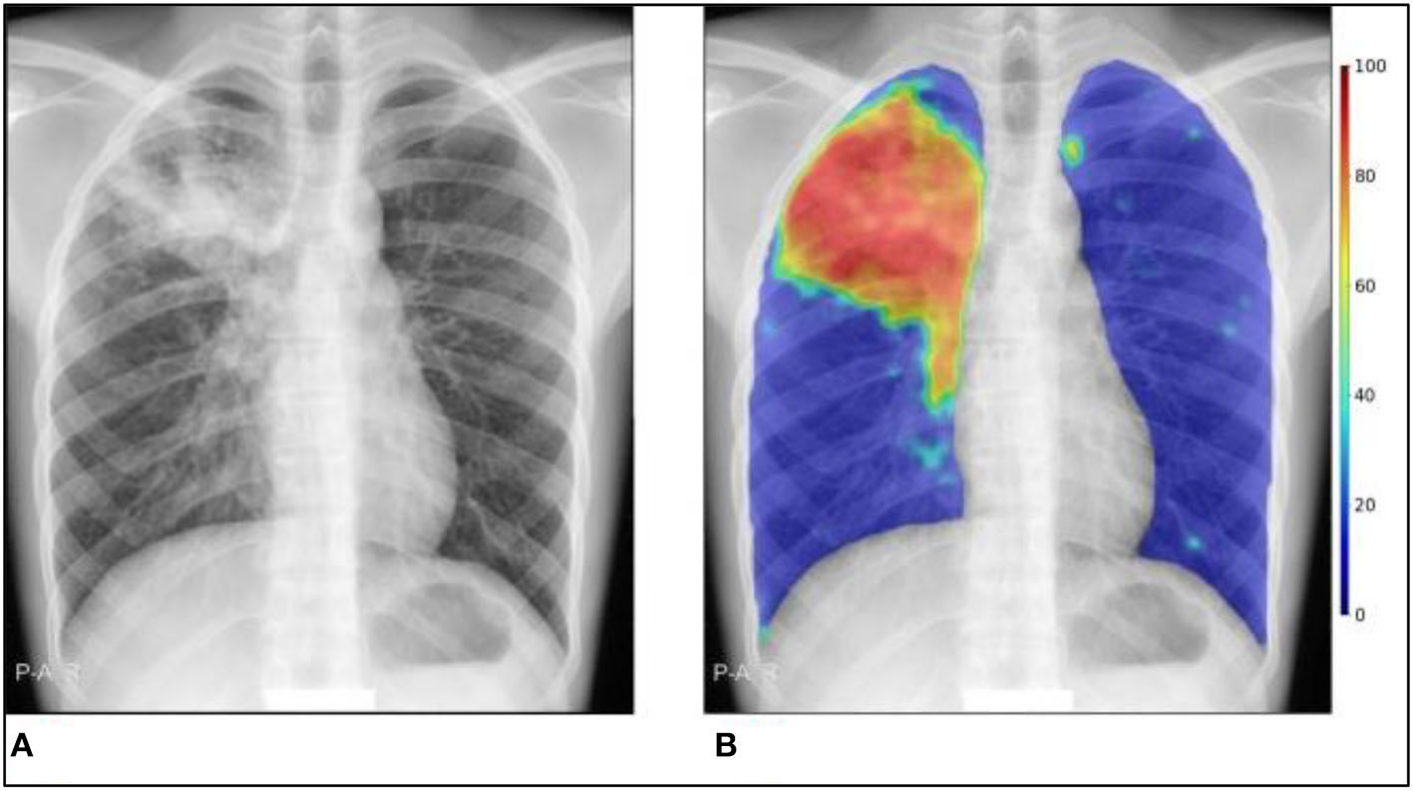
CAD4TB v6 output example. **(A)** Original CXR, **(B)** CXR with abnormality heatmap overlay. The Xpert test was positive, and the final composite CAD4TB score for this person was 91.7 (0 = normal, 100 = most abnormal).

Machine learning methods are used by the commercial software package CAD4TB to automatically identify TB from CXR pictures. Using separate annotated datasets, the software has been trained to recognize recognizable TB features in CXR pictures. It generates a number (0–100) that can be interpreted as the likelihood that the person has active TB that can be seen on CXR. Figure 2 shows an anomaly heatmap showing areas the software deems suspicious.

**Figure 3:**
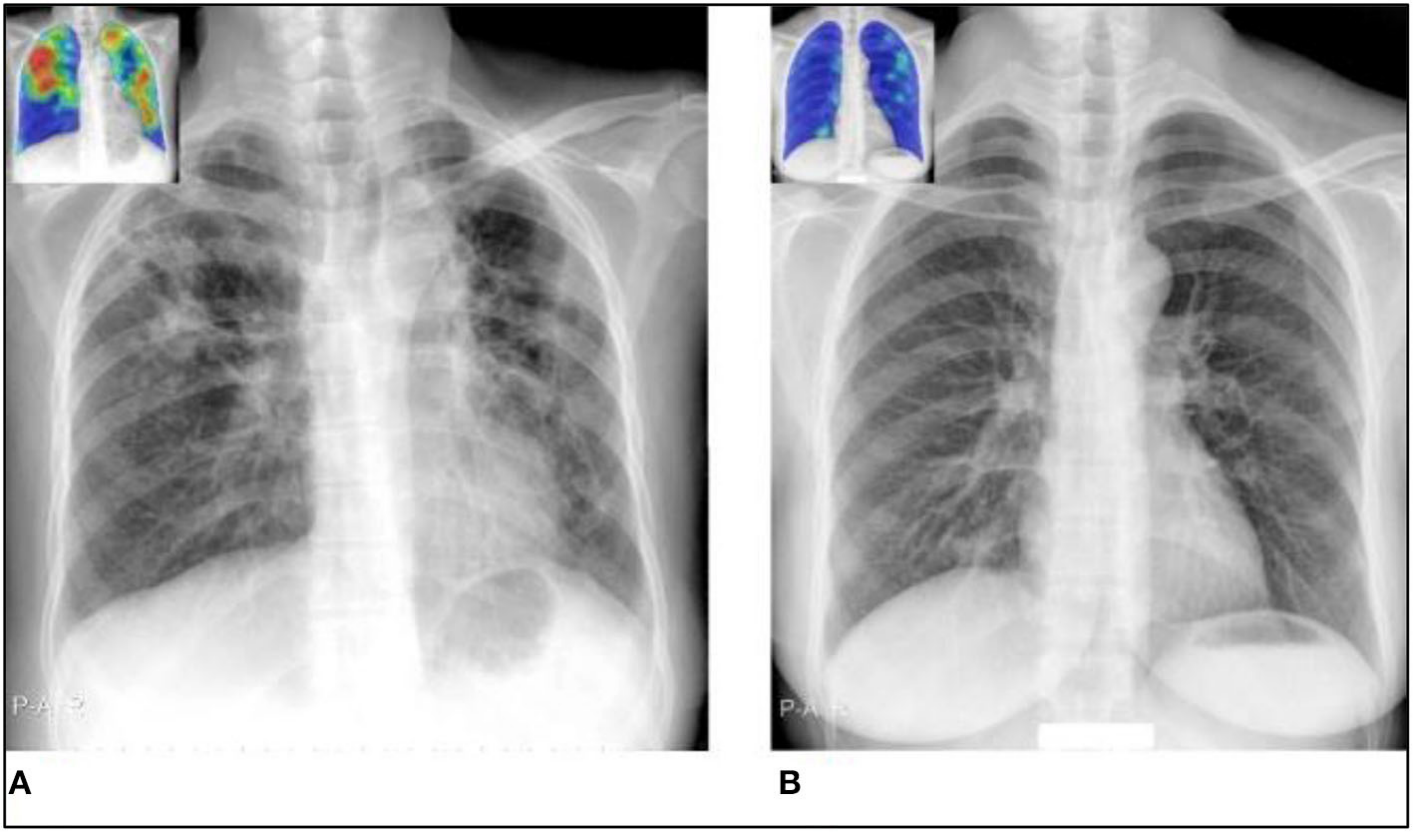
Situations in which the radiograph presentation does not match the geneXpert result, making radiograph-only prediction challenging for both observers and CAD4TB. The CAD4TB heatmaps are displayed in the inset photos, with blue denoting the majority of normal texture and red denoting the majority of abnormal texture. **(A)** A geneXpert-negative case identified by all five observers as TB positive (score 3) and by CAD4TB v6 (score = 100). **(B)** A geneXpert-positive case that received a score of 1 (no-TB) from four experts and a score of 2 from the final expert. When a sensitivity of 99% is reached, the 18.7 CAD4TB score for this case indicates that TB has not yet been detected.

Figure 3 shows a few instances in which the radiograph's appearance conflicts with the outcome of the geneXpert test. The first case shows a radiograph that appears consistent with tuberculosis. Still, the geneXpert test was negative, and the second case shows a normal radiograph, but the subject had a positive geneXpert test result. Both times, CAD4TB functions as anticipated and under-qualified observers. The causes of the discrepancy between these specific cases' geneXpert results and radiograph appearance are unknown.

**Figure 4:**
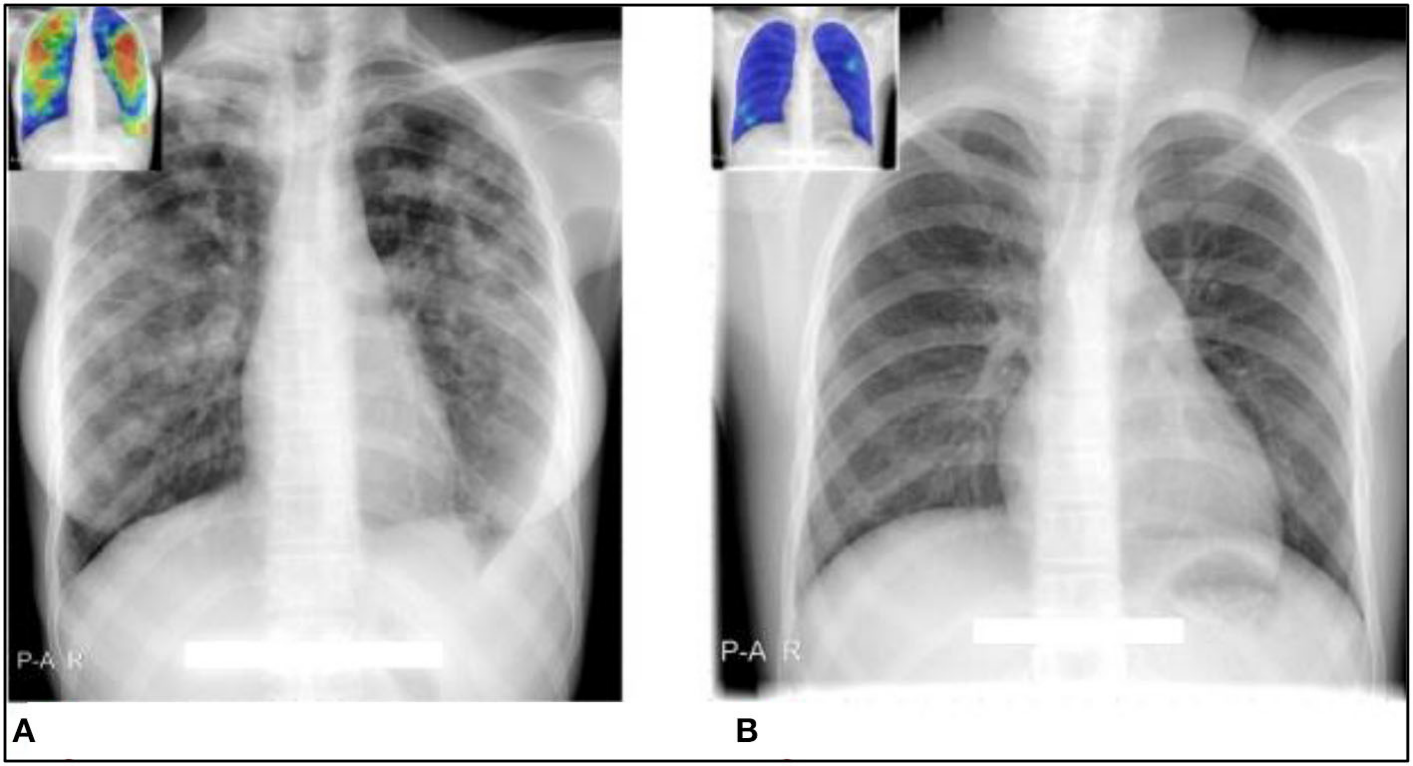
Cases where CAD4TB and observers' interpretations of radiographs agree well with geneXpert results. The CAD4TB heatmaps are displayed in the inset photos, with blue denoting the majority of normal texture and red denoting the majority of abnormal texture. **(A)** A case that tested positive for TB using geneXpert was rated as such by all five observers and CAD4TB v6 (score = 91.7). **(B)** A case that received a score of one (no-TB) from all five experts was geneXpert-negative. The case's CAD4TB score is 7.1.

In contrast, Figure 4 illustrates two simple situations where the results of the geneXpert and the radiograph (as evaluated by CAD4TB and radiologists) agreed.

## Discussion

To the best of our knowledge, this is the first study to evaluate the performance of the automatic software computer-aided detection for tuberculosis (CAD4TBv6) in diagnosing pulmonary TB on chest radiography and compare the CAD4TB software reading with the results of radiologists' reports in the Gaza strip, Palestine. Sputum samples were obtained from those with a score > 40 for GeneXpert analysis and those with symptoms strongly suggestive of pulmonary TB. A certified radiologist read the obtained CXRs.

Although the utilization of Xpert in programmed applications has increased recently, the WHO has also advocated using screening instruments like CXR that employ more affordable diagnostic algorithms (17, 29–32). Using an automated system to analyze a chest radiograph for the presence of active pulmonary TB produces objective, repeatable results and a consistent format for reporting. Creating software that provides automated CXR interpretation is a significant step toward connecting technology advancements to mass-screening initiatives for TB (23). In addition to increasing case identification in screening programs, using CAD4TB as a triage tool to pre-screen people for Xpert may help lower program expenses (33). Those with low CAD4TB scores had a low likelihood of testing positive for TB, so they might not be prioritized for Xpert testing using this method. Employing a triage tool such as CAD4TB might encourage more judicious use of Xpert by reducing the number of cartridges used in resource-constrained environments where there is not enough money to cover testing for all individuals with presumptive TB. This also applies to settings where onsite radiologists might not always be present to review CXRs. It is important to bear in mind that the costs associated with acquiring and operating digital X-ray devices must be balanced against the potential savings resulting from a reduced need for Xpert exams. This underscores the need for a comprehensive analysis that examines both the financial implications and consequences of widespread mass-screening through chest X-rays (CXR) (25).

According to research by Gautam et al. diabetes has been proven to increase both the likelihood of contracting TB and the severity of the illness (34). Additionally, research has demonstrated that smoking contributes significantly to the development of TB and raises the severity and fatality rates (35). However, our findings were in line with Tavaziva et al. as smoking and diabetes do not seem to make TB more likely to strike or to progress more severely (22).

According to 12 single-center assessment studies, the WHO estimates that Xpert's pooled sensitivity and specificity values for the detection of TB are 92.5 and 98%, respectively (36). However, the accessibility of digital radiography is a need for CAD utilization, which is not yet available in most resource-constrained low-burden settings. Nevertheless, it has been determined to be viable. It produces chest radiography significantly superior to traditional X-ray equipment in regions with low resources, such as Gaza Strip (37).

The findings of the current study of high specificity, high negative predictive value, high sensitivity, high diagnostic accuracy, and relatively low positive predictive value were consistent with findings from other studies in different settings testing CAD4TB (24, 25, 27, 38, 39). Compared to a confirmatory test like the Xpert, triage tests should have a sensitivity of 90% and a specificity of 70%, according to a 2014 WHO consensus meeting to define targets for new TB diagnostic technologies (40).

The findings of this study point to a feasible, effective, and even cost-effective strategy for TB screening in a symptomatic group that combines CAD4TB and symptomatology. Earlier research from different contexts are consistent with our findings (41–43).

The direct comparison between computerized and radiologist reading on the same set of pictures is one of the study's strengths. The extent to which this comparison may be generalized is severely constrained by the inter-reader variance in the reading of chest X-rays and the possibility of involving only one board-certified radiologist. The fact that our short-term study was only done in a single low-burden region presents a second limit that Data gathered from patients who presented with lower respiratory tract symptoms may not accurately reflect the prevalence of illness in the general community.

It is imperative that forthcoming research endeavors direct their attention toward comprehensively examining the multifaceted ramifications encompassing the adoption of Computer-Aided Diagnosis (CAD) within diverse low-burden nations. In particular, a rigorous investigation is warranted to elucidate the intricate interplay of financial, practical, and ethical considerations inherent in the deployment of CAD within these unique contexts. Subsequent investigative trajectories should encompass an exploration of the potential synergies arising from amalgamating CAD-generated outcomes with an array of clinical parameters, encompassing symptomatic manifestations and risk profiling. Furthermore, a profound research agenda should be undertaken to systematically assess the efficacy of CAD not only within the spectrum of operational feasibility but also across various technical dimensions indispensable for its seamless integration into prevailing diagnostic frameworks. Concurrently, an evaluative lens should be directed toward pioneering CAD products that are emergent within the market landscape. This comprehensive inquiry stands to provide a robust foundation for harnessing the maximal potential of CAD, charting its trajectory toward optimized medical diagnostics within the distinctive landscape of low-burden nations.

## Conclusion

Combining chest X-ray readings by CAD4TB and symptomatology is extremely valuable for screening a population at risk in a resource-limited country with a high burden of a neglected disease. CAD4TB is noticeably more efficient than other methods for TB screening and early diagnosis in people who would otherwise go undetected. In order to increase case finding and infection control and lower the cost of case detection within triage algorithms, CAD solutions may present an opportunity. This inspires further investigation into the best ways to utilize its potential as a support tool for clinical officers in the diagnostic interpretation of radiographs as well as a stand-alone triage test in systematic screening settings.

## Data availability statement

The raw data supporting the conclusions of this article will be made available by the authors, without undue reservation.

## Ethics statement

The study protocol was approved by the Palestinian Health Research Council (Helsinki Committee for Ethical Approval research number: PHRC/HC/1175/22). Additional approval was obtained from the included Gaza Strip hospitals. Study participants provided written informed consent for survey activities.

## Author contributions

SA: Conceptualization, Data curation, Formal analysis, Funding acquisition, Investigation, Methodology, Project administration, Resources, Software, Supervision, Validation, Visualization, Writing — original draft, Writing — review & editing. KZ: Methodology, Project administration, Software, Validation, Visualization, Writing — original draft, Writing — review & editing.
